# A graphical assessment of p-values from sliding window haplotype tests of association to identify asthma susceptibility loci on chromosome 11q

**DOI:** 10.1186/1471-2156-7-38

**Published:** 2006-06-14

**Authors:** Rasika A Mathias, Peisong Gao, Janet L Goldstein, Alexander F Wilson, Elizabeth W Pugh, Paulette Furbert-Harris, Georgia M Dunston, Floyd J Malveaux, Alkis Togias, Kathleen C Barnes, Terri H Beaty, Shau-Ku Huang

**Affiliations:** 1 Genometrics Section, Inherited Disease Research Branch, National Human Genome Research Institute, National Institutes of Health, Balitmore, USA; 2 Johns Hopkins Asthma and Allergy Center, Johns Hopkins University School of Medicine, Baltimore, USA; 3 Center for Inherited Disease Research, Johns Hopkins University School of Medicine, Baltimore, USA; 4 Department of Microbiology, Howard University College of Medicine, Baltimore, USA; 5 Department of Epidemiology, Johns Hopkins University Bloomberg School of Public Health, Baltimore, USA

## Abstract

**Background:**

Past work on asthmatic African American families revealed a strong linkage peak with modest evidence of association on chromosome 11q. Here, we perform tests of association for asthma and a panel of 609 SNPs in African American subjects using a sliding window approach. While efficient in screening a region of dense genotyping, this approach does create some problems: high numbers of tests, assimilating thousands of results, and questions about setting priorities on regions with association signals.

**Results:**

We present a newly developed tool, Graphical Assessment of Sliding P-values or GrASP, which uses color display to indicate the width of the sliding windows, significance of individual tests, density of SNP coverage and location of known genes that simplifies some of these issues, and use it to identify regions of interest in these data.

**Conclusion:**

We demonstrate that GrASP makes it easier to visualize, summarize and prioritize regions of interest from sliding window haplotype analysis, based jointly on the p-value from all the tests from these windows and the building of haplotypes of significance in the region. Using this approach, five regions yielded strong evidence for linkage and association with asthma, including the prior peak linkage region.

## Background

To search for causal genes for asthma on chromosome 11q, a region previously highlighted in linkage and association studies on African American families by our group, a dense panel of SNPs was genotyped using an Illumina™ fine mapping panel in these African American subjects. The sliding window approach that has gained much favor in recent years [1-6] to test for association between SNPs and haplotypes in this region was adapted to analyze case-parent trios and case-control data because it is a simple and efficient way to screen a region of dense genotyping. Given an ordered set of markers (1, 2, 3,....n), sliding windows of overlapping haplotypes are tested in sequence, i.e. markers 1-2-3 are treated as a single haplotype, then markers 2-3-4 are treated as a single haplotype, then markers 3-4-5, etc. Haplotypes of varying sizes (i.e. 2-, 3-, 4-SNP haplotypes, etc.) can be assessed. When large numbers of SNPs are used, this approach generates large numbers of non-independent statistical tests, and visualizing and interpreting these multiple tests can become daunting. Currently, it is common practice to use some form of summary measure (such as the minimum p-value of all sliding windows or windows of a particular size that include any one SNP) as a synopsis of each SNP. Here, we present a novel tool called Graphical Assessment of Sliding P-values or GrASP that provides a graphical overview of all tests from sliding windows without sub selection.

Asthma, a disease of chronic airway inflammation, shows strong familial aggregation [7-9] but models of inheritance are inconsistent, and linkage studies over the past decade have suggested that causal genes may be located on over two dozen chromosomal regions [10,11]. Heterogeneity in the genetic control of asthma is obvious from the variable evidence for linkage and/or association across studies and even among families within a single study. Recently, positional cloning studies have identified four novel candidate genes for asthma, including the genes encoding PDH finger protein 11 (PHF11) on chromosome 13q14 [12] dipeptidyl peptidase IV-related protein 3 (DPP10) on chromosome 2q14-q32 [13], a disintegrin and metalloproteinase domain 33 (ADAM33) on chromosome 20p13 [14], and G protein-coupled receptor for asthma susceptibility (GPRA) on 7p [15].

In a genome-wide scan and subsequent fine-mapping analysis, we have previously presented evidence of linkage of asthma to chromosome 11q markers in an African American population, but not Caucasian or Hispanic families [16,17]. Further, family based association and transmission disequilibrium tests (TDT) showed significant evidence of linkage and linkage disequilibrium for several individual markers in this region. A putative susceptibility locus was estimated to be at map position 70.8 cM with a confidence interval spanning the linkage peak [17]. Several candidate genes in this region were examined for association with asthma: *CRTH2*, which encodes a receptor for prostaglandin D_2 _(PGD_2_) [18]; FCER1B, encoding the high affinity IgE receptor β subunit [19]; and CC10, the Clara cell 10-kD immunomodulator [20]. Family-based and case-control analysis of asthma and two common SNPs (G1544C and G1651A) in the 3' untranslated region of *CRTH2 *showed significant evidence of linkage in the presence of disequilibrium in this African American and an independent Chinese population [21]. Here, we present further fine-mapping of this region on 11q in the African American families and apply GrASP to these data to illustrate its ability to summarize results from thousands of tests while identify regions of interest based on all single-SNP and haplotype tests of association.

## Results

Samples were genotyped at the SNP Center of the Genetic Resources Core Facility (GRCF) using the Illumina™ Fine Mapping Panel #38 spanning 52 Mb on chromosome 11q. The GRCF released genotypes on a total of 705 loci; the overall error rate for the entire data set was 0.55 % (considering duplicated samples – 46 events/8,410 genotypes) and the overall parent-child discordance rate was 0.07 % (4 events/5,612 genotypes). An additional 36 loci had minor allele frequencies < 5%, 8 had 50% Gencall scores < 0.40 [22], 18 markers had reproducibility between the duplicate samples (6 comparison pairs) of < 100%, 7 had Mendelian inconsistencies in families > 2%, and 43 showed significant departure from Hardy Weinberg Equilibrium (p-value < 0.01).

The final data for the family-based analysis included 86 probands, 63 fathers and 75 mothers from the 89 nuclear families resulting in 60 child-father pairs, 73 child-mother pairs or 51 complete trios. The case-control analysis for replication included the 86 probands and 94 control samples. A total of 609 markers on 318 samples were used in the analyses, with an average distance between SNPs of 86119 bp (range: 127 – 3676182 bp). Average linkage disequilibrium (LD) between neighboring SNPs was modest with an average D' = 0.53 and average r^2 ^= 0.11.

### Single SNP results

A total of 48 SNPs had p-values < 0.05 in the case-control data and 34 in the family data (6 of which were significant in both data sets). Table 1 presents results for these 6 common SNPs, and for 20 SNPs that had p-values < 0.01 in either the case-control or family data, along with the known genes where they are located.

**Table 1:** Summary of single SNP results from case-control- and family-based tests.

SNP	Case-Control p-value	Family-based p-value	SNP location (Build 34)	Gene
rs8187	0.4902	0.0006	35721356	FLJ10774
rs2001176	0.0136	0.0012	35921461	ABTB2
rs952489	0.4769	0.0078	36584163	
rs17640	0.0017	0.0896	36803905	
rs1323855	0.0064	0.0039	37320740	TRIM44
rs1822934	0.0132	0.0396	38997664	
rs1026604	0.1875	0.0090	41508291	
rs8929	0.0688	0.0072	47006838	SYT13
rs1447567	0.0050	0.5271	47431052	
rs501738	0.8875	0.0039	59919843	
rs734777	0.0070	0.2252	60505067	
rs1675090	0.0076	0.3620	64453906	
rs1212146	0.0300	0.0173	66928565	NRXN2
rs1783734	0.0788	0.0095	68013303	
rs1540209	0.0263	0.0237	70451445	SUV420H1
rs1017639	0.0007	0.1615	71121458	
rs1249579	0.0092	0.5639	71294225	
rs873860	0.1812	0.0090	72273439	TMEM16A
rs624765	0.0031	0.7237	72480733	PPFIA1
rs534668	0.3425	0.0016	74816006	STARD10
rs519790	0.1869	0.0000	74836264	STARD10
rs1791926	0.0030	0.5864	75282569	P2RY2
rs1320646	0.0127	0.0290	78697013	GARP
rs948763	0.0049	0.2763	81161079	LOC440057
rs1151188	0.0077	0.4141	81385674	
rs586607	0.4162	0.0012	84860384	

### Selecting regions for haplotype analysis

Considering 609 SNPs and sliding window sizes from 2 – 6 SNPs per window, there are 3030 non-independent tests for each study design (608 2-SNP windows, 607 3-SNP windows, 606 4-SNP windows, 605 5-SNP windows and 604 6-SNP windows) for a total of 6060 tests. Hence, our first approach was to construct systematic rules based on prior information about LD and/or single SNP results to create blocks or segments of contiguous SNPs within which sliding window-based tests could be conducted. The density of SNPs in this fine mapping panel was not optimal for establishing patterns of high LD or constructing large haplotype blocks; although there were 16 blocks of LD identified using the approach of Gabriel et al. [23], most were very small (11 had only 2 SNPs per block, 3 had 3 SNPs, and one each of 5 and 6 SNPs per block). The physical distance across the blocks was also modest (14 were under 25 Kb, one at 136 Kb and only one at 486 Kb). To restrict haplotype tests with sliding windows within these haplotype blocks would have ignored much of the data available here.

Therefore, we began with those single SNPs with marginal levels of significance and expanded outward from these to define segments of contiguous SNPs over which to implement sliding window haplotype analysis. There were 76 SNPs that had a positive signal (p-value < 0.05) in either the case-control or family-based analysis. As a first step, we created a mini-segment including all SNPs within a 1 Mb region centered around each of these 76 SNPs (i.e. 500 Kb on either side). In the next step, these 76 mini-segments were merged wherever two segments overlapped. This resulted in 15 contiguous main segments (covering 498 SNPs), with 15 gaps (covering 111 SNPs) between segments (where no evidence against the null hypothesis was seen). We then performed sliding window haplotype tests within each segment but not across segments, which resulted in 2268 tests for each study design.

### Sliding window haplotype results

**Figure 1:**
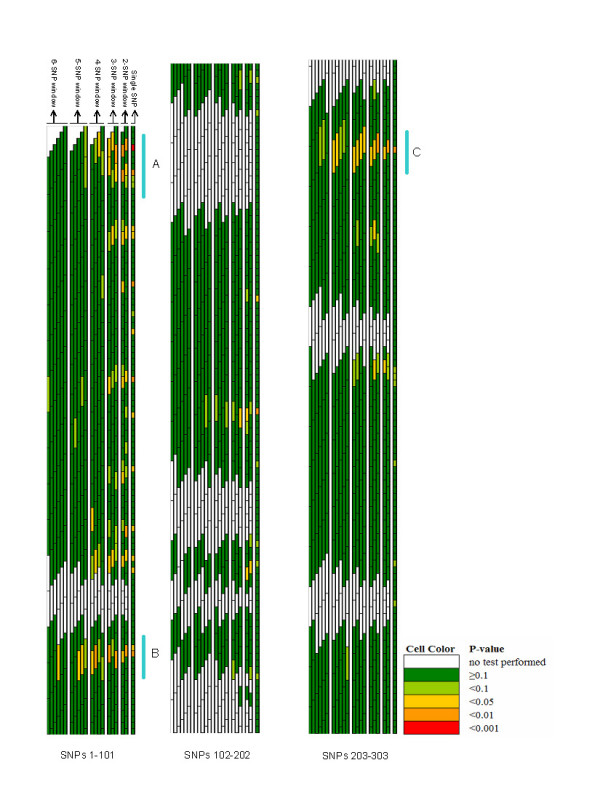
Graphical Assessment of sliding p-values for all tests performed on SNPs 1–303 in the family-based data with prioritized regions of association signal (labelled A-C).

**Figure 2:**
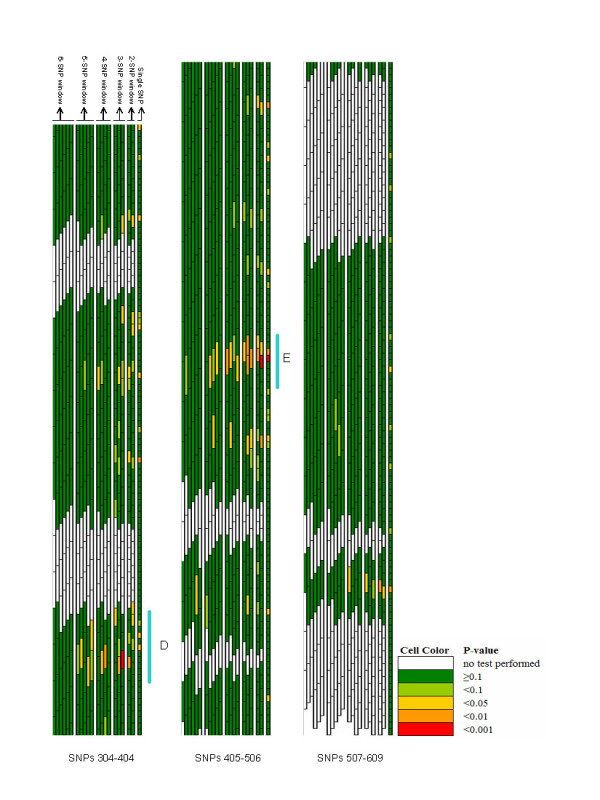
Graphical Assessment of sliding p-values for all tests performed on SNPs 304–609 in the family-based data with prioritized regions of association signal (labelled D-E).

Figures 1 and 2 are a compact graphical overview of all tests performed on the family data. These figures represent p-values from single SNP TDT tests and overall p-values from haplotypes constructed across sliding windows of sizes 2–6. Each test is represented by a single tile: a box with a black outline which is as long as the number of SNPs in the window, and the p-value of the test is represented by the color of the box. Several important points become immediately apparent and are discussed below.

Examining p-values for any one SNP (i.e. considering the minimum p-value across all windows that included a SNP), reveal many SNPs had significant results (79 with minimum p-value <0.05, 25 <0.01 and 5 <0.001). Prioritizing regions of interest for further follow up is not a simple task, especially since the minimum p-value should be weighed against the number of tests yielding significant results when setting priorities. GrASP provides a visual presentation of the results that can simplify the identification of regions with both high significance and where multiple tests yielded high significance. Here, we consider regions of highest priority as those that have a building of windows with significance across multiple SNPs.

Relying upon these patterns, five regions depicted in Figures 1 and 2 (and labelled A – E) stand out as "regions of interest". Each of these regions contain SNPs with minimum p-values that are statistically significant, multiple windows in the region showing significance, and have a pattern of building from smaller to larger windows until the strength of apparent association drops off for the largest haplotypes (as would be expected considering the breakdown of the original putative causal haplotype over physical distance).

**Figure 3:**
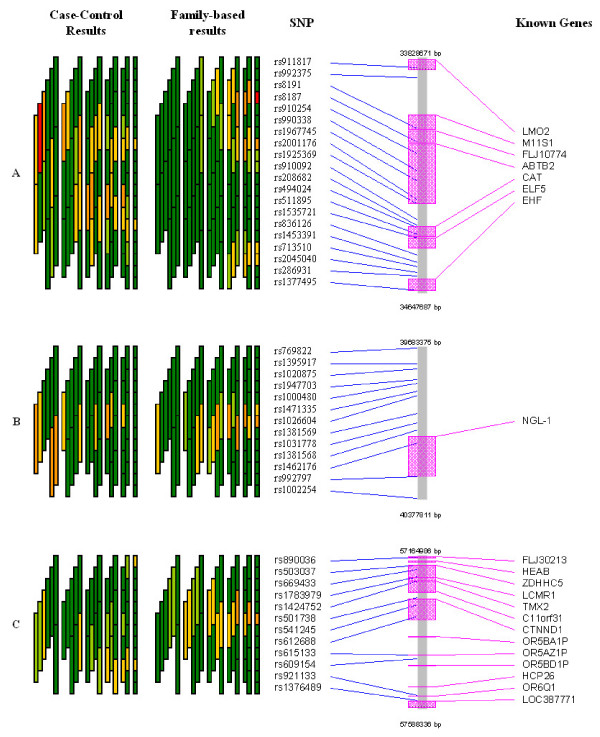
Detailed overview of the first three regions with strongest association signals (regions A-C in Figure 1) for case-control- and family-based tests along with SNP and known gene locations. Color coding of p-values are as presented in Figure 1.

**Figure 4:**
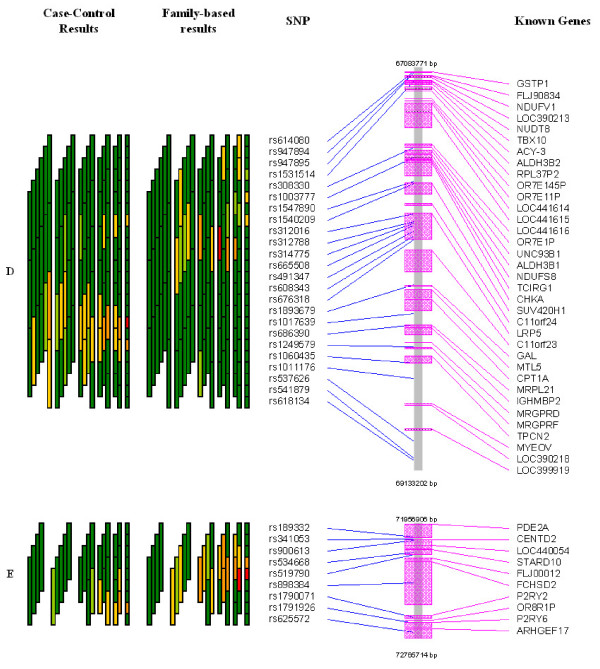
Detailed overview of the final two regions with strongest association signals (regions D-E in Figure 2) for case-control- and family-based tests along with SNP and known gene locations. Color coding of p-values are as presented in Figure 1.

Figures 3 and 4 present these five regions (labelled A-E) in more detail, including evidence from the case-control analysis, the physical location of SNPs and known genes in this region (both features available in GrASP – either relying on user-provided input or by querying public databases of Entrez). Examining the details of chromosomal regions using this approach can simplify the process of identifying regions of association and prime candidate genes. The five regions with strong patterns in the family data also had signals of association in the case-control data, although the two did not always align perfectly.

## Discussion

In this report we present a graphical tool for haplotype association analysis utilizing data of dense SNPs on chromosome 11q for asthma in an African American population, and identify five regions that may include susceptibility loci for asthma.

High throughput genotyping and dense SNP maps makes the sliding window approach for analyzing haplotypes appealing, and several groups have explored this approach from a statistical [24-29] and applied perspective [1-6,30]. In this sliding window approach, windows of varying size are examined, generally beginning with the first SNP and moving windows down the framework map one SNP at a time. This strategy is simple and efficient in comprehensively screening a dense region of genotyping for association with the trait of interest. This approach, however, does raise problematic issues: exceedingly high numbers of non-independent tests, the task of assimilating thousands of test results, attempting to create haplotypes across SNPs far from each other, and prioritizing "regions of interest" based on sometimes weak signals from these multiple tests. Here, we provide a novel graphical simplification to some of these issues in our implementation of the Graphical Assessment of Sliding P-values (GrASP) analysis tool.

The use of graphical presentations and has proved extremely useful in other analytical strategies for mass amounts of data, including linkage analysis [31], linkage disequilibrium patterns [32], identification of haplotype block structure [23], and most commonly in high-throughput gene expression profiling (e.g. microarray) [33-38]. In many of these diverse applications, color representation can quickly reveal underlying patterns. The use of sliding haplotype windows yields high numbers of tests; in this study sliding windows of 2–6 SNPs over 609 SNPs yielded a maximum of 3030 tests. Limiting regions of sliding windows can reduce the number of tests, but summarizing 2268 tests to identify patterns of association is still difficult. Although using a summary measure, such as the minimum p-value for each SNP, can simplify this issue, it would be ideal to have all test results available for assessment without selecting sub-sets. Here too, as in the case of LD and gene expression patterns, a graphical overview (as implemented in GrASP) is both practical and effective.

The simple assimilation of all the results itself (seen in Figures 1 and 2) overcomes the task of presenting 2268 test results simultaneously, and clearly shows most of the regions have no evidence of statistically significant signals (i.e. green background). However, there are several regions with distinct signals (yellow, orange and red cells), but only a handful of these are compelling (defined by clusters of yellow, orange and red cells).

Setting priorities for regions of association from case-control or case-parent trio data as used here is a key step in identifying putative asthma and atopy genes in this region on chromosome 11q. For any single SNP, up to 21 tests of association are performed (one single SNP test, two 2-SNP windows that include the SNP, three 3-SNP windows, four 4-SNP windows, five 5-SNP windows, and six 6-SNP windows that include the SNP) using this sliding window approach. Relying solely upon minimum p-value for each SNP from these tests clearly ignores the number of tests performed. Graphically examining the strength of the signal for each SNP (the minimum p-value) and the number of tests providing evidence at this SNP as part of increasingly larger haplotypes using GrASP presents both types of information jointly (Figures 1 and 2). Currently one can weigh the evidence in priority regions informally, but the application of formal rules (including aspects such as effect size and haplotype frequencies) could be implemented. Based on this approach, 5 regions can be identified as priorities for subsequent mapping, but our prioritization scheme is not meant to rule out the few additional regions with smaller signals (see Figures 1 and 2). How to narrow the putative region of interest is another major question, and we show that this issue can also be simplified with GrASP. Here, by searching for increasingly significant haplotypes in the areas of interest (as depicted in Figures 3 and 4), the location of the strongest signals given the current genotyping density is easy to determine. This provides the investigator a reasonable starting point for additional genotyping, particularly for SNPs to be typed within and flanking a current block of association.

Finally, as we were unable to use haplotype blocks to guide our haplotype analyses, we substantially reduced the number of sliding window tests by relying on our single SNP results. In these data, all strong signals from the sliding windows were centered at or around a SNP with an individually significant association. However, considering situations with very few single-SNP signals or extremely dense SNP data with high LD, our rules for forming segments (within which sliding window tests are done) may discard too much or too little data. Furthermore, limiting haplotype tests in regions highlighted by single SNPs tests may introduce several issues: missing regions of association that would only have been detected with a haplotype test (i.e. those for which the single-SNP tests did not have sufficient power), and perpetuation of false positives especially when limiting haplotype tests only to SNPs in tight LD with those single SNPs yielding a significant association. Considering issues such as these, it is more appropriate that the sliding window approach should consider the density of SNPs at hand first analyzing smaller windows and then proceeding to larger windows where there are signals from the smaller windows rather than restricting windows to regions based on single-SNP results. Given our SNP density (here relatively sparse for fine mapping), it appears that the windowing approach should be implemented only for windows of size 2–4. The 5- and 6-SNP windows should be tested only where the smaller windows reveal positive associations. This in itself would have reduced the number of tests by an additional 864 (38%) for each analysis type. GrASP now makes this strategy to reduce the number of tests for sliding window approach easy to implement, and thereby efficient.

The five regions of priority contain many known genes as illustrated in Figures 3 and 4. Although most of these genes are not immediately recognized as candidates in the pathophysiology of asthma and atopy, a few are notable. Two genes (ELF5 and EHS) from region A encode members of the ETS transcription factor subfamily [39], show epithelial-specific expression, and their gene products may act as a transcriptional repressors. These two genes have been suggested as candidate genes for asthma in a genome-wide linkage analysis in Tristan da Cunha [40,41], and variants of the EHS gene were shown to be weakly associated with asthma susceptibility in a Caucasian case-control study [42,43]. The gene LRP5 in region D encodes the low density lipoprotein receptor-related protein 5 and has been linked to the susceptibility to osteoprosis and IDDM [44]. It is important to note that the peak fine-mapping marker, D11S1337, is situated within this locus [17]. Interestingly, about 400 kb away from LRP5, the TCIRG1 (gene which encodes a vacuolar proton pump H(+)-ATPase) is also included in a region of significant association. Two different gene products through alternative splicing have been found for TCIRG1, one of which, TIRC7, encodes a protein essential in T-cell activation, and is almost exclusively expressed in the immune system [45].

## Conclusion

In conclusion, we have demonstrated that the use of GrASP can serve as a step towards simplifying multiple issues in the sliding window approach for haplotype analysis: summarizing test results, setting priorities for defining regions of interest and narrowing these regions of interest. Here, GrASP is used on fine-mapping SNP data, but it may also assist greatly in assessing SNPs within candidate genes of interest and even more so in genome-screen SNP data analysis. We have identified five regions with strong evidence for association with asthma in these African American subjects which contain many known genes including a few noteworthy candidates. Most compelling, is the strong evidence from region four that also includes our peak linkage marker reported previously.

## Methods

### Study population

Study protocols for the Collaborative Study on the Genetics of Asthma (CSGA) were approved by the Institutional Review Boards of Johns Hopkins University School of Medicine and Howard University College of Medicine. Written informed consent was obtained from each of the study subjects. A total of 89 African American case-parent trios were included[17,21]. The ascertainment scheme and clinical characteristics of these families have been described in detail elsewhere [16]. Briefly, families were ascertained through an asthmatic sib-pair and asthmatic probands in case-parent trios. In the case-control design, 89 unrelated individuals were selected from among asthmatic probands of 49 multiplex African American families and 40 case-parent trios. In addition, 95 randomly selected healthy individuals were included as a replication study in a case-control study design. We should note that while our sample size is limited, these data are a subset of the original families that provided compelling evidence for linkage and association in this region of 11q, and therefore a good sample for fine-mapping.

### Genotyping

Genetic analysis was conducted on either genomic DNAs or DNA amplified directly from blood using multiple displacement amplification (MDA) technology (Molecular Staging Inc.) [46,47]. Genotyping was performed at the SNP Center of the Genetic Resources Core Facility (GRCF) at the McKusick/Nathans Institute of Genetic Medicine, Johns Hopkins School of Medicine. Genotypes were generated on a BeadLab 1000 system [22] using the Illumina™ Fine Mapping Panel #38 (Illumina OPA ID = FM78) spanning 52 Mb on chromosome 11q (35–87 Mb – using the Build 34 map).

### Statistical methods

At the single-SNP level, the family-based association test (FBAT) as described by Rabinowitz and Laird [48] with the empirical variance-covariance estimator was used for the family-based sample and in the case-control design, χ^2 ^tests were used to test for independence between single SNPs and disease status.

For the haplotype analysis, sliding windows of 2 – 6 SNPs each were considered as the approach allows for comprehensive screening of the whole region of genotyping. In the family-based analysis, haplotype tests were performed using methods described by Horvath et al [49] which assumes no recombination between markers, and is analogous to the single marker multi-allelic statistic described by Rabinowitz and Laird [48] allowing for weighting all phased genotypes (haplotypes) possible in the family. An EM algorithm which maximizes the likelihood of the phased haplotype frequencies based on all observed family genotypes and computed under the null hypothesis was used to estimate haplotype frequencies used to obtain these weights. For the case-control data, group-wise haplotype frequencies were estimated using an EM algorithm [50]. χ^2 ^values were calculated for each haplotype versus all others, and empiric significance was assessed using 10,000 permutations of the observed case-control data. The "omnibus test" was evaluated to detect differences in overall haplotype frequency profiles between asthmatics and controls [50]. For both the family- and case-control analyses only the overall test statistic was considered, i.e. one single overall test per sliding window, and not the individual tests of deviation from expected for each haplotype.

### Graphical overview of sliding windows

Graphical Assessment of Sliding P-values or GrASP, a tool to graphically present and assess p-values from sliding window haplotype tests, was implemented. This program can summarize thousands of p-values from sliding window tests of variable sizes in a simple graphic that uses varying levels of a user-specified color to indicate the width of the sliding windows and the level of statistical significance attained. With this tool it now becomes a simple task to identify regions/blocks of interest from these sliding windows, based jointly on the absolute p-value of the tests and the building of haplotypes of significance in the region. GrASP is executed as an Excel macro, and is written in Excel's built-in version of Visual Basic for Applications. It uses as input, a summary file that contains the test results stored in simple format (SNP order, first SNP of each window and p-values from windows of varying sizes) that is easily constructed from analysis output considering the repetitive nature of the output results. GrASP also illustrates SNP location and the location of known genes in the region as a physical track that is drawn either using user-provided input, or by querying the public databases of Entrez. GrASP is freely available for use at: .

## Authors' contributions

RAM conceived of the graphical method, participated in the acquisition of data and family recruitment, performed statistical analysis and wrote the manuscript. PG carried out part of the molecular genetic studies and helped draft the manuscript. JLG did all the programming for GrASP and helped draft the manuscript. AFW and EWP helped design GrASP and helped draft the manuscript. PFH, GMD, FJM and AT participated in the design of the study and recruitment of families for the study and helped draft the manuscript. KCB and THB participated in study design, recruitment of families for the study, guidance on statistical analysis and helped draft the manuscript. SKH was involved in study design, molecular genetic studies and helped draft the manuscript. All authors read and approved the final manuscript.
